# IL-33-induced alternatively activated macrophage attenuates the development of TNBS-induced colitis

**DOI:** 10.18632/oncotarget.15984

**Published:** 2017-03-07

**Authors:** Lei Tu, Jie Chen, Dandan Xu, Zhongming Xie, Bing Yu, Ying Tao, Guixiu Shi, Lihua Duan

**Affiliations:** ^1^ Division of Gastroenterology, Union Hospital, Tongji Medical College, Huazhong University of Science and Technology, Wuhan, Hubei Province, China; ^2^ College of Medicine, Xiamen University, Fujian, China; ^3^ Department of Rheumatology and Clinical Immunology, The First Affiliated Hospital of Xiamen University, Xiamen, Fujian, China

**Keywords:** IL-33, inflammatory bowel disease, macrophage, M2, Arginase-1, Immunology and Microbiology Section, Immune response, Immunity

## Abstract

Accumulated data have shown that alternatively activated macrophage exerts a modulatory role in many diseases, including colitis. Interleukin-33 (IL-33), a critical modulator in adaptive and innate immune, has been implicated in autoimmunity and inflammation. Previously, we have reported that IL-33 functions as a protective modulator in TNBS-induced colitis, which is closely related to a Th1-to-Th2/Treg switch. Here, we present novel evidence suggesting that IL-33 primes macrophage into alternatively activated macrophages (AAM) in TNBS-induced colitis. The strong polarized effect of IL-33 was tightly associated with the markedly increased induction of Th2-type cytokines. To confirm the beneficial effects of AAM induced by IL-33, peritoneal AAMs isolated from IL-33-treated mice were transferred to recipient mice with TNBS colitis. The adoptive transfer resulted in prominent inhibition of disease activity and inflammatory cytokines in the TNBS-treated mice. In conclusion, our data provide clear evidence that IL-33 plays a protective role in TNBS-induced colitis, which is closely related to AAM polarization.

## INTRODUCTION

Inflammatory bowel disease (IBD), including Crohn's disease (CD) and ulcerative colitis (UC), is a chronic intestinal inflammatory condition that is medicated by genetic, immune and environmental factors [1, 2]. The pathophysiology and etiology of IBD are not fully understood and the treatment options for these diseases are quite limited. To dissect the mechanism and develop novel therapeutic strategies for IBD, various kinds of animal models have been established [3]. Trinitrobenzene sulfonic acid (TNBS)-induced experimental colitis characterized by a predominant Th1/Th17-mediated immune response and mucosal inflammation which closely resembles important immunological and histopathological aspects of CD [4]. In a previous study, we also showed an increased expression of IL-17 and IFN-γ in colonic tissue and sera in TNBS-induced murine colitis [5].

Macrophages are key innate immune cells in orchestrating the host innate and adaptive immune responses, which are implicated in engulfing infectious microorganisms and tissue debris, and presenting antigens to T cell and B cell [6]. In several human diseases, a crucial role in the resolution of tissue injury and promotion of tissue repair acted by macrophages was described [7, 8]. Typically, macrophages are classified into two main groups: classically-activated macrophage (CAM, M1) and alternatively activated macrophage (AAM, M2). CAMs is generated in response to Th1-related cytokines (IFN-γ, TNF-α), while AAM polarization is linked to the Th2-related cytokines (IL-4 and IL-13) [9]. By secreting inflammatory cytokines such as TNF-α and IL-6, CAMs exhibit anti-microbial activities and promote Th1 cell responses. Conversely, AAMs produces fewer pro-inflammatory cytokines and plays an important role in the resolution of inflammation and tissue remodeling and repair [8]. In different inflammatory conditions, it is worth noting that macrophage can be polarized into distinct subpopulations, depending on the specific immune mechanisms during disease pathogenesis.

IL-33 has been identified as a novel member of IL-1 family, synthesized as a 30-kDa precursor protein and could be cleaved by caspase-1 forming a 18-kDa mature protein [10]. Previous studies have shown that IL-33 binds its specific receptor ST2L, which is selectively expressed on Th2 cells but not Th1 cells [11, 12]. Due to different micro-environmental cues, it has been reported that IL-33 plays important and complicated roles in many human diseases. In the Th2-mediated allergy, IL-33 plays a deleterious role associated with the activation and production of type II cytokines and AAM polarization [13, 14]. However, by switching Th1/Th17 to Th2 type immune response, IL-33 can reduce the development of atherosclerosis [15], graft rejection and EAE [16, 17], which are mainly mediated by Th1 and Th17 response.

Our previous data have shown that in TNBS-induced colitis rIL-33 treatment led to a striking improvement in both the clinical and histopathological aspects of the colitis, which is dependent on Treg expansion via up-regulating CD103+ DCs [5]. In the present study, we sought to dissect the role of IL-33 in de novo generation of AAM during the development of colitis. Our data here suggest that AAM could be essential for inflammation recedes and tissue repair in TNBS-induced colitis by IL-33 administration, and this process is tightly controlled by Th2-type cytokines.

## RESULTS

### IL-33 induces AAM in TNBS-induced colitis

**Figure 1:**
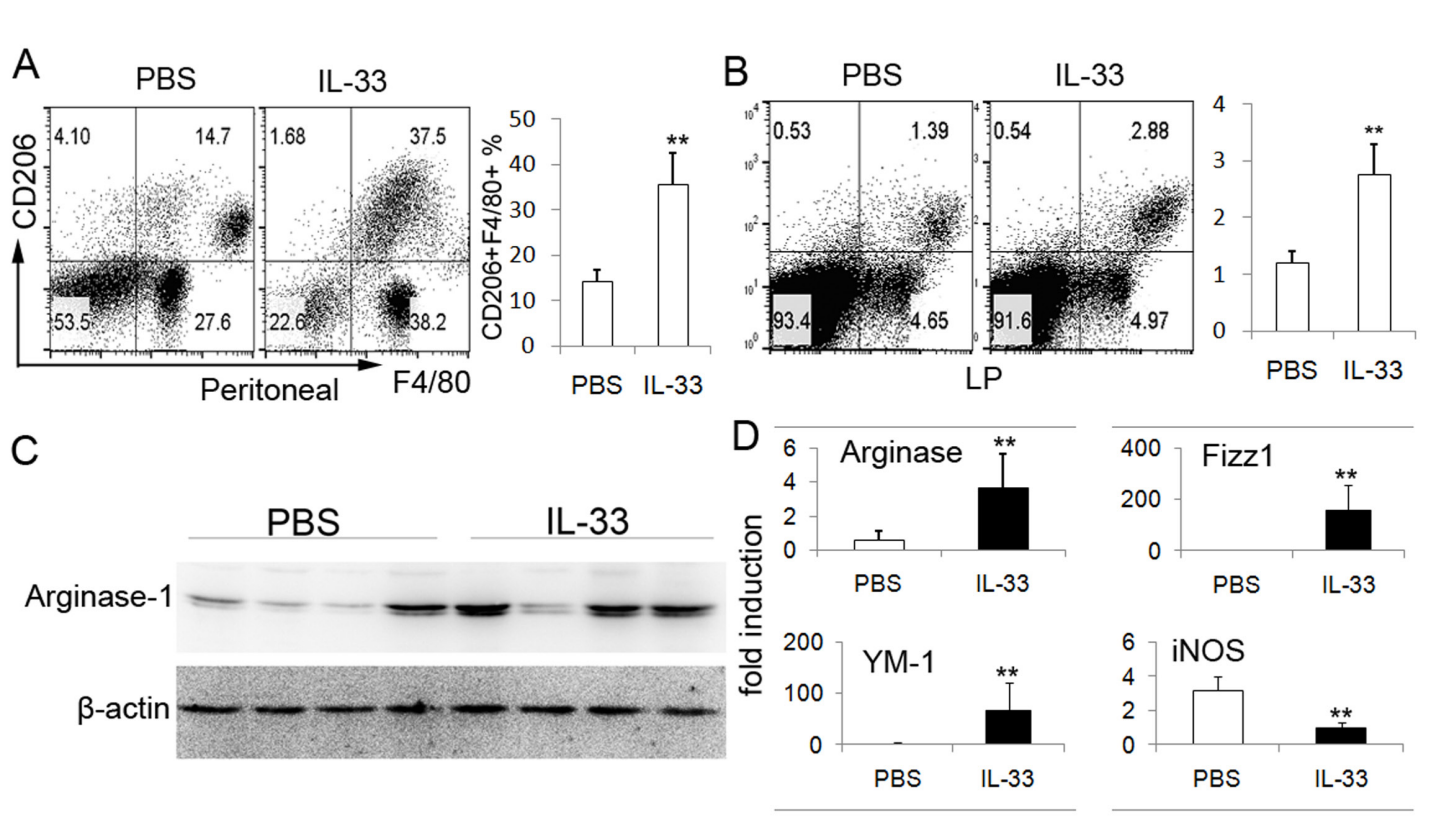
IL-33 treatment increases the number of AAM in TNBS-induced colitis Peritoneal cells and LPMCs were collected from the colitis mice treated with IL-33 or PBS on day 4 after administration of TNBS. Peritoneal cells **A**. and LPMCs **B**. were analyzed by flow cytometry for F4/80 and CD206. The data shown are representative one of three separate experiments. **C**. Colonic tissues of TNBS-treated mice were collected to analyze the Arginase-1 expression by immunoblot. The data are presented of four mice investigated. The results shown are one from out of three independent experiments. **D**. Total mRNA was extracted from colonic tissues to analyze the expression of Arginase-1, Fizz1, YM-1 and iNOS by real-time PCR. Data represent means ± SD of each group (*n* = 6 - 8/group). * *p* < 0.05, ** *p* < 0.01.

In our recent study, an increased expression of IL-33 has been demonstrated in TNBS-induced mice colitis. The reason for this might be that the endogenous IL-33 in the colonic tissues is induced by the inflammatory response raised. To further determine the role of IL-33 in TNBS-induced colitis, the regulatory macrophage was investigated. IL-33 or PBS was administrated into mice daily starting at the time of TNBS administration through intraperitoneally injection. Compared with PBS treated group, IL-33-treated mice displayed markedly elevated proportion of F4/80+CD206+ double positive cells in peritoneal cavity (Figure 1A), which were considered as AAM. Accompanied with increased AAM in peritoneal cavity, a specific abundance of F4/80+CD206+ double positive cells was observed in the lamina propria (LP) of colonic tissues from colitis mice with IL-33 treatment (Figure 1B). To further confirm the AAM polarization by IL-33 in TNBS-induced mice, the markers of AAM were detected. Arginase-1, the relatively specific protein in AAM, was strongly up-regulated in IL-33-treated mice (Figure 1C). These data suggest that the IL-33 promotes AAM polarization in TNBS-treated mice.

### IL-33 alters cytokines profile in TNBS-induced colitis

**Figure 2:**
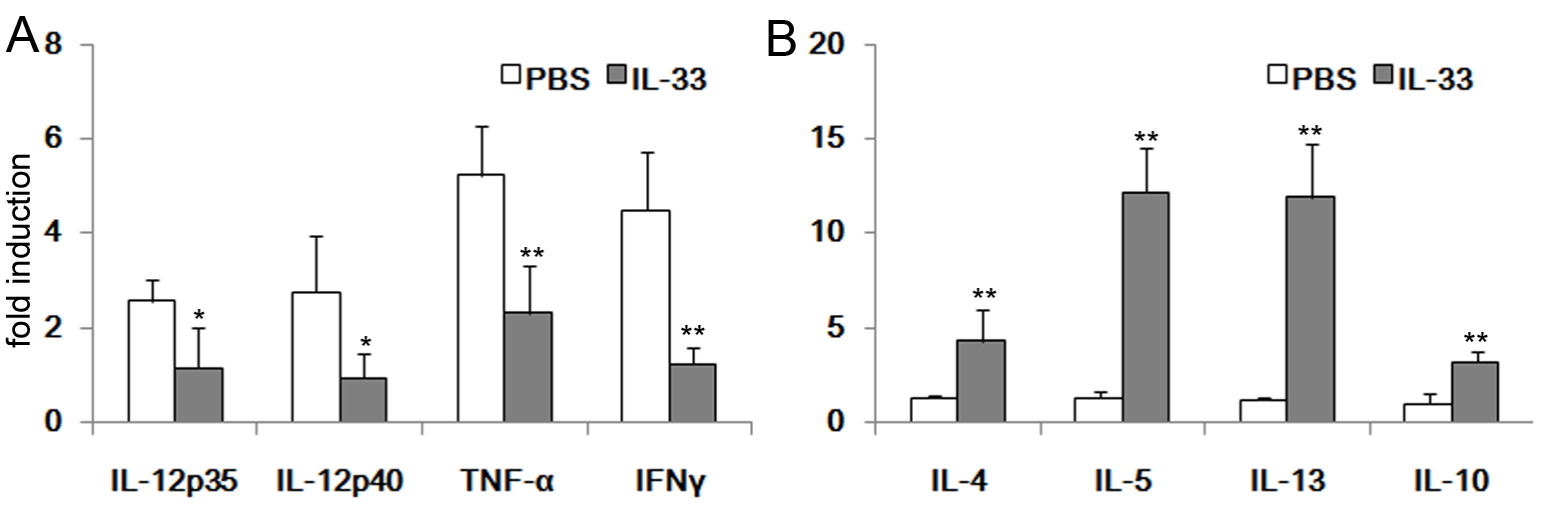
RT-PCR analysis of cytokines mRNA in the colonic tissues Colonic tissues of TNBS-treated mice with IL-33 or PBS treatment were collected at day 4 after administration of TNBS. Total RNAs were prepared and subjected to real-time RT-PCR analysis of IL-12p35, IL-12p40, TNF-α, IFN-γ, IL-4, IL-5, IL-13 and IL-10. Results represent means ± SD (*n* = 6 - 8/group). * *p* < 0.05, ** *p* < 0.01.

To further ensure the beneficial effect of IL-33 in the development of TNBS-induced colitis, CAMs are generated in response to Th1-related cytokines (IFN-γ, TNF-α), while AAM polarization is linked to the Th2—related cytokines (IL-4 and IL-13). The total mRNA of colonic tissues was harvest to evaluate the expression of genes of interest. To our expectation, IL-12, IFN-γ and TNF-α expressions were significantly decreased in the mice with IL-33 treatment (Figure 2A). Conversely, IL-4, IL-5 and IL-13 were increased. In addition, it is worth noting that the key regulated gene, IL-10, was also highly expressed in IL-33 treated mice (Figure 2B).

### IL-33 enhances the polarization of AAM dependent *in vitro*

**Figure 3:**
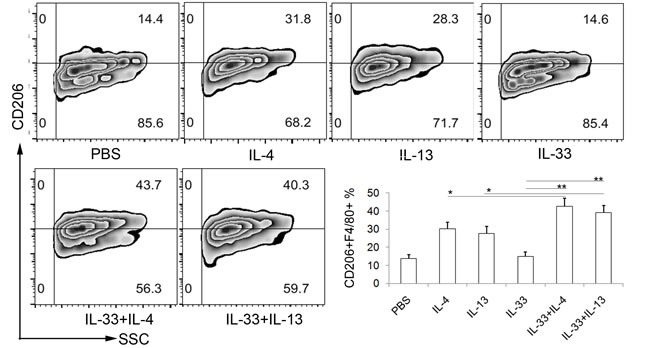
IL-33 indirectly promotes AAM development ***in vitro***. BMMs were differentiated as described in materials. The differentiated BMMs were activated by IL-4 (10 ng/ml), IL-13 (10 ng/ml) and IL-33 (20 ng/ml) alone, or by IL-33 in combination with IL-4 or IL-13 for 48h, followed by flow cytometry analysis of CD206 expression. The F4/80 positive cells were gated for analyzing. Data are from one experiment representative of three. Results represent means ± SD (*n* = 6 - 8/group). * *p* < 0.05, ** *p* < 0.01.

Considerding the expression of ST2L on macrophages and the well-known contribution of IL-33 to type 2 immune responses, we reasoned that IL-33 may be involved in AAM polarization. To determine whether IL-33 directly induces the differentiation of AAMs, we generated macrophage cells from bone marrow cells of mice, and then cultured them with IL-33, IL-4, IL-13 alone or in combination. The proportions of AAM were analyzed by flow cytometry. As expected, IL-4 and IL-13 had apparent effect on the expansion of AAM (Figure 3A). No significant increase of AAM in IL-33 treatment alone was observed. However, it is worth noting that IL-33 synergize with IL-4 or IL-13 to enhance AAM polarization (Figure 3B).

### IL-33 promotes AAM polarization *in vivo*

**Figure 4:**
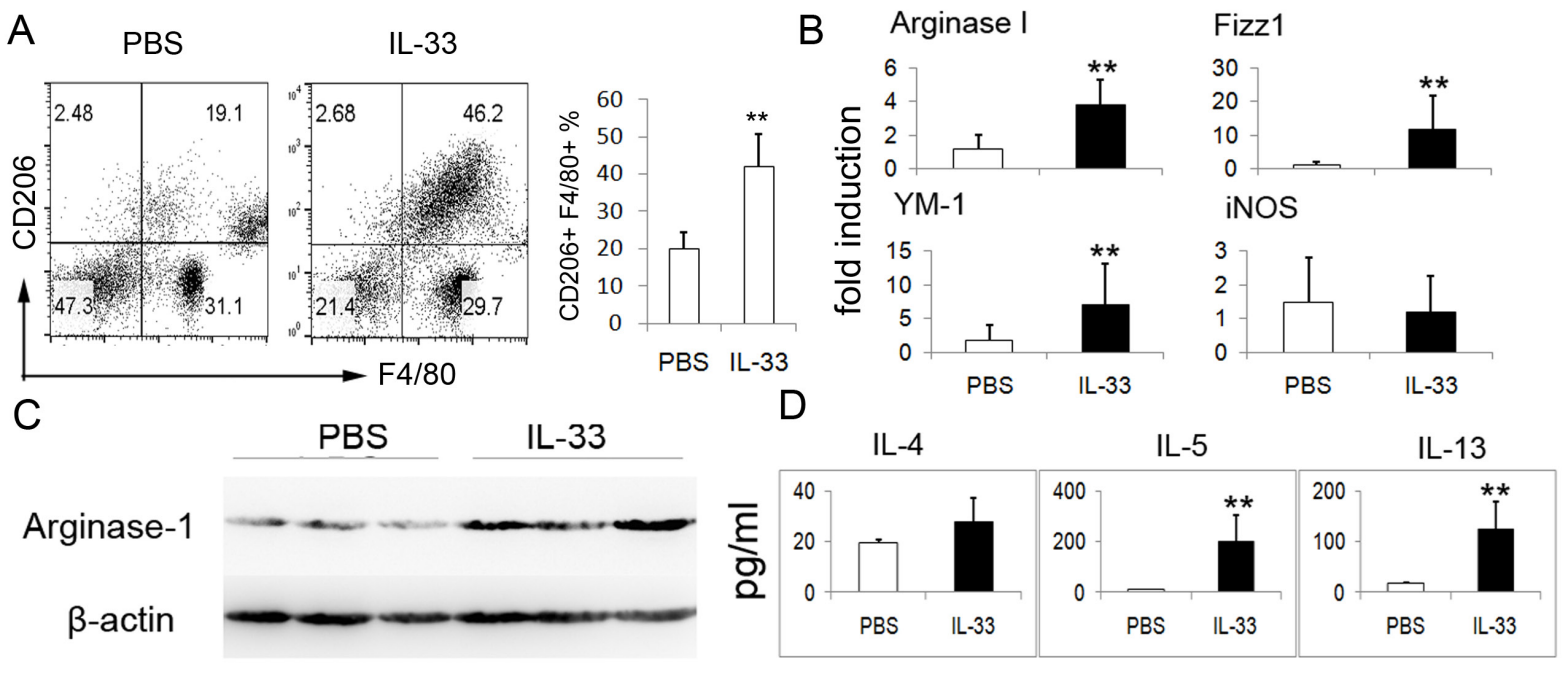
IL-33 promotes AAM polarization ***in vivo***. To confirm the effect of IL-33 in the development of AAM, BALB/c mice (*n* = 4) were administrated intraperitoneally (i.p.) with IL-33 (2 ug)or PBS daily. Peritoneal cells and colonic tissues were extracted from normal mice treated with rIL-33 or PBS on day 3. **A**. CD206 expression on peritoneal F4/80+ macrophages was analyzed by flow cytometry. The data shown are representative. **B**. Arginase-1, Fizz1, YM-1 and iNOS mRNA of colonic tissues were assessed by quantitative real-time PCR. Results represent means ± SD. **C**. The expression of Arginase-1 protein in colonic tissues was assessed by immunoblot. **D**. Peritoneal lavage fluids from mice were harvested, IL-4, IL-5, IL-13 levels in the lavage fluids were determined by ELISA. The data shown are representative one of three separate experiments. ** *p* < 0.01.

Above data showed that IL-33 strongly enhanced the AAM polarization in the presence of IL-13 or IL-4, we then addressed the role of IL-33 in priming macrophage to AAM *in vivo*. For this purpose, exogenous IL-33 was i.p. injected into mice, mice received same amount of PBS were served as controls. The AAM in the peritoneal cavity were investigated. As expected, IL-33-treated mice exhibited markedly increased number of AAM in peritoneal cavity as compared with PBS controls (Figure 4A). Besides, IL-33-treated mice displayed highly increased mRNA expressions of AAM-related genes including FIZZ1, Arginase 1 and YM1 (Figure 4B). Consistently, an increased expression of Arginase-1 protein in colonic tissues of IL-33-treated mice was also observed by immunoblots (Figure 4C). In this study, a considerable increase of IL-4, IL-5 and IL-13 in peritoneal lavage fluid of mice with IL-33 treatment were detected (Figure 4D).

### Transfer of IL-33-induced AAM reduces colitis

**Figure 5:**
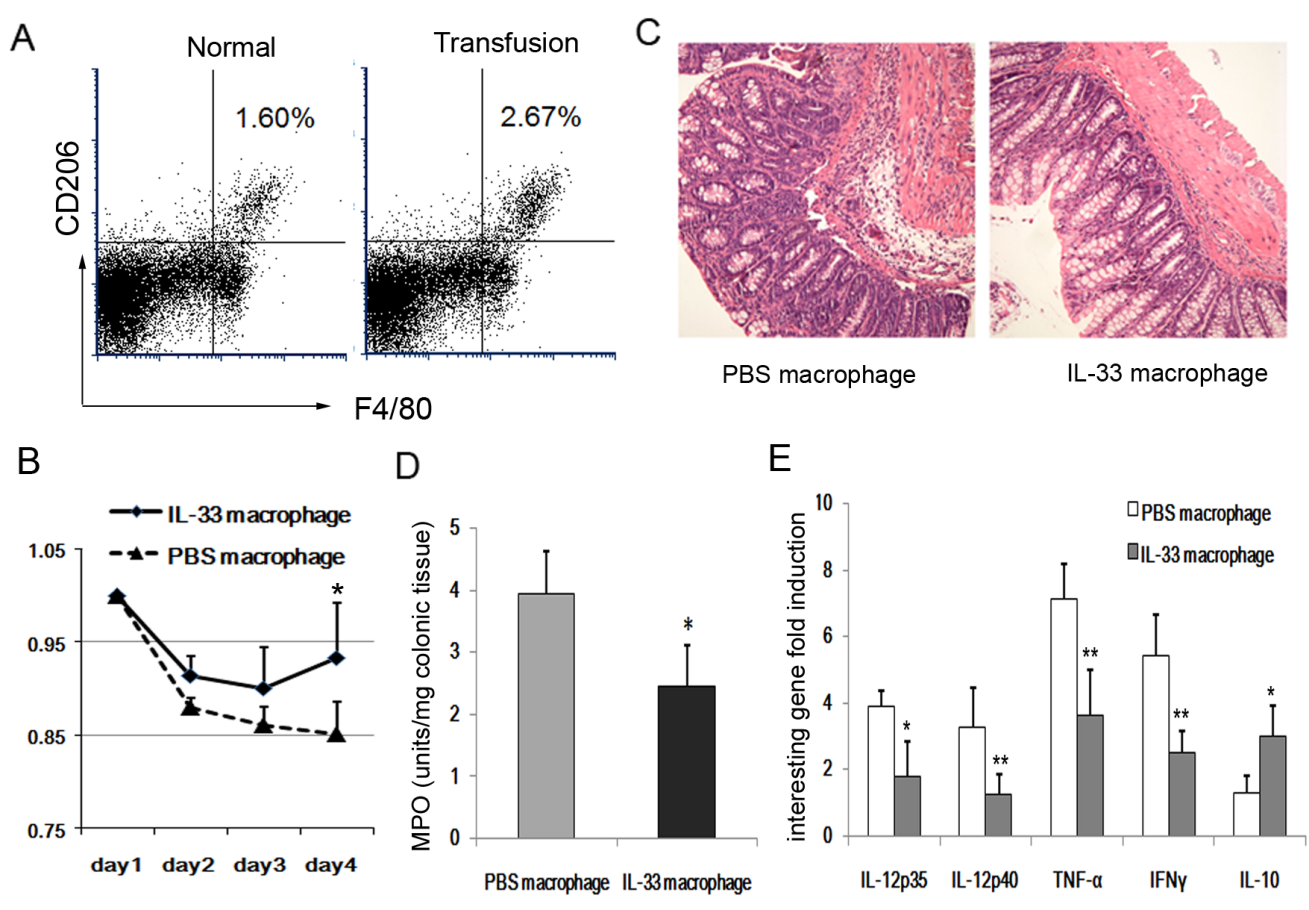
Introperitoneal infusion of IL-33-induced AAM attenuates the development of colitis Normal mice were injected IP with IL-33 or PBS and 3 days later sacrificed. The peritoneal macrophage were harvested and transferred into mice 48 hours prior to TNBS instillation (*n* = 6 - 8/group). **A**. Mice with IL-33-induced AAM transfusion or not. After 24h injection, the LPMCs were harvest and subjected to analyze the AAM through CD206 and F4/80 double staining. **B**. The variations BW of original weight were recorded on day 4 following the TNBS inoculation. **C**. Histological analysis of colonic tissues after TNBS inoculation revealed a reduced leukocytes infiltration in IL-33-induced AAM group as compared to that of PBS group. **D**. Colonic MPO levels was measured as described in materials. **E**. IL-12p35, IL-12p40, TNF-α, IFN-γ and IL-10 mRNA of colonic tissues were assessed by quantitative real-time PCR. Results represent means ± SD. Data are representative of three independent experiments. * *p* < 0.05, ** *p* < 0.01.

To directly examine anti-inflammatory properties of IL-33-induced AAM in murine colitis with TNBS instillation, normal mice were injected IP with IL-33 or PBS and 3 days later the peritoneal macrophage were harvested and transferred into mice 48 hours prior to TNBS instillation. The AAM in intestinal tissues of mice before and after IL-33-induced AAM transfusion were determined. Adoptive transfer with IL-33-induced AAM significantly increased the number of AAM in intestinal tissues (Figure 5A), and the AAM exerted a protective role in body weight loss during the development of colitis (Figure 5B). In addition, histochemistrical assay showed that there was decrease amount of infiltrated inflammatory cells (Figure 5C). The activity of MPO was decreased in the mice with IL-33-treated macrophage transfer (Figure 5D). Furthermore, the mRNA expressions of inflammatory cytokines IL-12p35, IL-12p40, IFN-γ and TNFa were markedly down-regulated in colonic tissues, whereas IL-10 was increased in mice transferred with IL-33-induced AAM when compared with PBS-treated macrophage (Figure 5E).

### Inactive CD patients show an increased Arginase-1 expression

**Figure 6:**
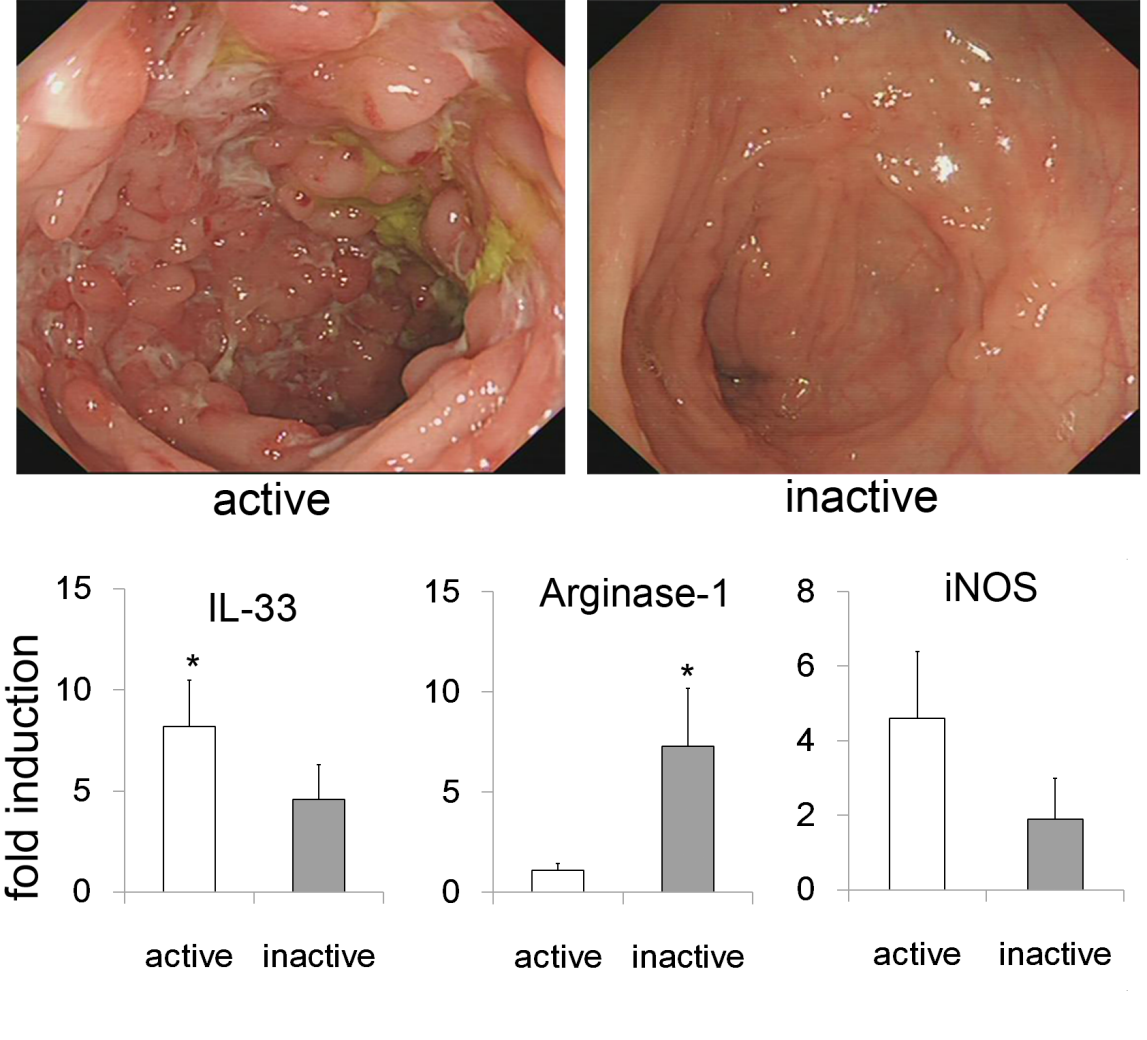
Argianse-1 expression in biopsy specimens from CD patients Endoscopic imaging showed the changes of inactive and active CD patients. The biopsy specimens were harvested from inactive (*n* = 6) and active CD patients (*n* = 8), and subjected to analyze the IL-33, Arginase-1 and iNOS expression by quantitative real-time PCR. Data are means ± SD. * *p* < 0.05.

To explore the relevance of our findings to clinical disease, the colonic biopsies of active and inactive CD patients were collected. As shown in Figure 6, CD patients with inactive disease appeared and no ulceration and inflammatory hyperemia by endoscopic evaluation. In line with the findings in our animal study, the levels of IL-33 and arginase-1 mRNA were markedly elevated in inactive CD patients, and a decreased tendency of iNOS. These results suggest that AAM might contribute to the inflammation remission and tissue repair.

## DISCUSSION

Previous findings have revealed a protective role for IL-33 in the development of TNBS-induced colitis via promoting a switch from intestinal Th1 to Th2/Treg responses [5]. Here, our results provide evidences for a novel mechanism by which IL-33 ameliorates the intestinal inflammation via priming macrophage towards alternatively activated macrophage. We observed that although IL-33 alone had no apparent effect on the AAM differentiation, IL-33 significantly promotes the AAM polarization in the presence of IL-13 or IL-4. Data presented in our studies demonstrate that IL-33 is a key modulator during innate and adaptive immune responses in the development of IBD. Although an increased IL-33 expression was elicited by the colonic inflammation, we think that IL-33 exerts as a negative feedback mechanism in inflammation caused by TNBS [3, 18].

Heretofore, studies have shown that IL-33 may have a very complex role in the pathophysiology of diseases. A crucial effect of IL-33 has been described in asthma [19]. The genes encoding IL-33 and ST2/IL1RL1 have been identified as major susceptibility loci for human asthma in childhood [20]. Additionally, IL-33/ST2 contributes to Ag-induced airway inflammation and plays a significant role through priming AAM polarization and promoting chemokine production [13]. Recent studies also indicate that IL-33 induces production of large amounts of IL-5 and IL-13 by group 2 innate lymphoid cells (ILC2s) [21–23]. In our study, an elevated IL-13 and IL-5 expression was also detected, which might be produced by ILC2 [24, 25]. IL-5 and IL-13 are important cytokines in the asthma by recruiting eosinophil and neutrophil. On the contrary, in the development of atherosclerosis, IL-33 markedly increased the levels of IL-4, IL-5, and IL-13, and decreased the level of IFN-γ [15]. Interestingly, in Th1/Th17-mediated EAE and rheumatoid arthritis, a protect role of IL-33 was observed in EAE through promoting AAM polarization and inhibiting Th1/Th17 differentiation [16], while IL-33 exhibit an adverse effect in rheumatoid arthritis via enhancing Th1/Th17 immune response [26]. Due to the different milieu of diseases, the different functions of IL-33 were performed in immune response that acts by binding to the orphan receptor ST2L.

Indeed, IL-33/ST2 axis also plays a dichotomous role in IBD pathogenesis. Previous studies have also demonstrated that increased expression of IL-33 plays a pathological role in the development of IBD, which showed a specific increase of mucosal IL-33 in active UC [18]. In the mice model of IBD, ST2 gene KO mice showed a significantly improved signs of colitis, and IL-33 treatment impaired epithelial barrier permeability *in vitro* and *in vivo* [27]. However, our previous study found a significant beneficial effect of IL-33 on Th1/Th17-mediated experimental colitis, and a deleterious consequence with anti-IL-33 antibody although without reaching significance [5]. The beneficial effect was associated with a prominent induction of Treg cells. In this study, we further confirmed the protective role of IL-33 in the development of TNBS-induced colitis. We demonstrated here that the protective effects of IL-33 are also resulted from AAM polarization and suppressive cytokine IL-10. Interestingly, IL-33 may be involved in the pathogenesis of DSS-induced acute colitis by promoting Th2 cell response in intestinal mucosa of mice [28], while IL-33 alleviates DSS-induced chronic colitis in C57BL/6 mice colon lamina propria by suppressing Th17 cell response as well as Th1 cell response [29]. Furthermore, IL-33 signaling protects from murine oxazolone colitis by supporting intestinal epithelial function, and IL-33 promotes IgA production to maintain gut microbial homoeostasis and restrain IL-1α-dependent colitis [30]. In this study, we also provided evidence for a novel mechanism by which IL-33 ameliorates the intestinal inflammation via priming macrophage into alternatively activated macrophage. All in all, IL-33 plays a complicated role in SAMP1/YitFc (SAMP) mice, DSS-induced colitis, oxazolone (OXA) colitis and TNBS-induced experimental colitis, which might be a result from discrepancy of dominated immune response.

Analysis of inflammatory and antiinflammatory pathways in the intestine of IBD patients provides therapeutic methods. The excessive production of inflammatory Th1- and Th17-related cytokines in CD tissue has been described in many reports, Th2-related immune response induced by several cell types was involved in the negative regulation of inflammatory pathways in the gut. TNF-α and IFN-γ were the key player in the pathogenesis of CD, which can prime macrophage to pro-inflammatory CAM [9]. The macrophage is adaptable, and its phenotype/function is critically determined by the micro-environment. Conversely, macrophage can also be activated by IL-4 and IL-13, which lead to activation of anti-inflammatory AAM. AAMs produce anti-inflammatory molecules and various components of extracellular matrix and contribute to the host response to tissue repair and healing [8]. It has been reported that IL-25-mediated anticolitic effect is associated with the induction of AAMs [31]. Interestingly, worm infection has been realized as a treatment of colitis. Infection with the worm Hymenolepis diminuta reduced the severity of dinitrobenzene sulfonic acid (DNBS)-induced colitis in mice [32]. Subsequent analysis revealed that H diminuta infection resulted in increased intestinal number of AAM. In our study, we also observed the expression of AAM markers, such as arginase-1 and FIZZ1, was significant elevated in IL-33-treated mice. The same results were also depicted in EAE and allergy mice treated with IL-33 [13, 16]. Furthermore, transfer of AAM to mice attenuates TNBS Colitis and EAE. Here, we also observed that transfer of IL-33-induced AAM contributed to the sustaining the mucosal inflammation in TNBS-induced colitis.

In conclusion, our studies here demonstrate that IL-33 treatment substantially ameliorates the development of TNBS-induced experimental colitis by priming macrophage to AAM. In combing with our previous study, we represent strong evidence that IL-33 might offer an alternative therapeutic method in managing CD.

## MATERIALS AND METHODS

### Human subjects and animals

Patients diagnosed with CD were recruited from the outpatient clinic of the division of Gastroenterology, Union Hospital, Tongji Medical College, Huazhong University of Science and Technology. Endoscopic biopsies were obtained from 8 CD with active disease, and 6 with inactive disease. Patients were between 27 and 55 years and with a mean age of 40 year. There was no difference between active and inactive patient in the gender, age. Biopsies from patients with inactive disease showed no ulceration or inflammation noted in endoscopic visualization or histologic analysis. All studies involving human tissue were approved by the Ethics Committee at the Tongji Medical College, Huazhong University of Science and Technology. The informed consent was obtained from patients and control subjects before the study. The methods were carried out in accordance with the approved guidelines. Female BALB/c mice were purchased from the Institute of Experimental Animal, Chinese Academy of Medical Sciences (Beijing, China). Six to eight wk-old female mice (body weight 20-30 g) were used for the study. The mice were bred and maintained in a specific pathogen-free barrier facility. All of the studies were performed in accordance with the Xiamen University Animal Care and Use Committee guidelines. The experimental procedures and the animal use and care protocols were approved by the Xiamen University Animal Care and Use Committee.

### Antibodies and reagents

Expression and purification of mouse recombinant IL-33 were carried out as previously described. Fluorescently labeled anti-F4/80 and anti-CD206 were purchased from Biolegend (San Diego, CA, USA). TNBS solution was obtained from Sigma-Aldrich. The primary antibodies rabbit anti-Arginase-1 and rabbit anti-β-actin were purchase from Santa Cruz Biotechnology.

### *In vivo* study

TNBS-induced colitis was performed as described elsewhere. Mice received control PBS or rIL-33 (2 μg/day) diluted in PBS daily by intraperitoneally injection at the time of TNBS administration until day 4. In the adoptive transfer model, normal mice were injected IP with IL-33 or PBS and euthanized 3 days later. The peritoneal macrophages were harvested and transferred into mice 48 hours prior to TNBS instillation. The lamina propria mononuclear cells (LPMCs) and peritoneal cells were harvested as described in previous study [5]. Hematoxylin/eosin staining was employed to assess the pathological changes of sections derived from colonic tissues.

### BMM differentiation

Bone marrow-derive macrophage (BMM) was generated from bone marrow cells in the presence of M-CSF as reported in previous study [33].

### ELISA

Peritoneal lavage fluid collected from the normal mice which injected IP with IL-33 or PBS. The levels of IL-4, IL-5, and IL-13 cytokines were determined by ELISA kits (ebioscience) according to the manufacturer's instructions.

### Immunoblots

Colon protein extraction and immunoblot assay was performed as described previously [34]. The primary antibody was incubated at 4 °C overnight, followed by staining with secondary antibody conjugated horseradish peroxidase. The immunoreactivity was evaluated by an ECL system (Pierce).

### SYBR green real-time RT-PCR

Colonic tissues were harvested and subjected to RNA isolation using the TRIzol (Invitrogen) reagent according to the manufactures's instruction. cDNA was synthesized from 2 μg mRNA using a first strand DNA synthesis kit (Fermentas life sciences). PCR reaction mixture was prepared using SYBR Premix Ex Taq (TaKaRa) according to the manufacturer's instructions. Relative expression levels for cytokines were normalized by GAPDH and calculated by using the 2^−ΔΔCt^ method. Primer sequences used for IL-12p40, IL-12p35, Arginase-1, Fizz1, YM1 and iNOS are listed in Table 1. The primers for IL-4, IL-5, IL-13, IL-10, IFN-γ, and TNF-α gene were previously described [5]. The expression of each cytokine in normal mice was used as calibrator.

**Table 1:** Primer sequences for qRT-PCR

mouse	IL-12 p40 sense	GGAAGCACGGCAGCAGAATA
	IL-12 p40 antisnese	AACTTGAGGGAGAAGTAGGAATGG
	IL-12 p35 sense	CCTCAGTTTGGCCAGGGTC
	IL-12 p35 antisnese	CAGGTTTCGGGACTGGCTAAG
	Arginase-1 snese	GTCTGGCAGTTGGAAGCATC
	Arginase-1 antisnese	TGGTTGTCAGGGGAGTGTTG
	Fizz1 sense	CCAATCCAGCTAACTATCCC
	Fizz1 antisense	TGGTCCAGTCAACGAGTAAG
	YM1 sense	CATGAGCAAGACTTGCGTGAC
	YM1 antisense	GGTCCAAACTTCCATCCTCCA
	iNOS sense	CAGCTGGGCTGTACAAACCTTC
	iNOS antisense	CATTGGAAGTGAAGCGTTTCG
human	Arginase-1 sense	TTGGCAATTGGAAG-CATCTCTGGC
	Arginase-1 antisense	TCCACTTGTGGTTGTCAGTGGAGT
	IL-33 sense	CAAAGAAGTTTGCCCCATGT
	IL-33 antisense	AAGGCAAAGCACTCCACAGT
	iNOS sense	GGCTCAAATCTCGGCAGAATC
	iNOS antisense	GGCCATCCTCACAGGAGAGTT
	β-actin sense	AGAAAATCTGGCACCACACC
	β-actin antisense	AGAGGCGTACAGGGATAGCA

### Flow cytometry

The LPMCs and peritoneal macrophage were incubated with fluorescence-conjugated mAbs in staining buffer. Abs used for flow cytometry were described in *Antibodies and reagents*

### Measurement of myeloperoxidase (MPO) activity

MPO activity was assessed as a marker for neutrophil leukocyte infiltration into the inflamed colon tissue, and was determined as previously described [35]. Values are expressed as MPO units per gram of wet tissue.

### Statistical analysis

Data are presented as mean ± SD. Group comparisons were performed using Student's t test by GraphPad Prism software. *p* values (two-tailed) below 0.05 were considered as significant.
